# Diagnostic Value of Symptom Screening for Pulmonary Tuberculosis in China

**DOI:** 10.1371/journal.pone.0127725

**Published:** 2015-05-22

**Authors:** Jun Cheng, Lixia Wang, Hui Zhang, Yinyin Xia

**Affiliations:** Chinese Center for Disease Control and Prevention, Beijing, China; Kliniken der Stadt Köln gGmbH, GERMANY

## Abstract

**Objective:**

To evaluate the diagnostic value of symptom screening for tuberculosis (TB) case finding defined in National Tuberculosis Control Program in China (China NTP) among elderly people(≥65 years) and younger people(<65 years).

**Methods:**

We made a secondary analysis in a population-based TB prevalence survey in China in 2010. Questionnaire including information for cough and haemoptysis was completed by face to face interview, and then chest radiography was conducted in all eligible participants. Sputum smear and culture were followed for all TB suspects. We calculated the odds ratios (OR), sensitivity, specificity, positive predictive value (PPV), negative predictive value (NPV) and the area under the receiver operating characteristic curve (AUC) of using different symptoms for screening to detect bacteriologically positive TB in subpopulations stratified by age 65, to evaluate the performance of symptom screening for TB.

**Findings:**

Of 315 newly diagnosed bacteriologically positive TB, 131 patients (41.59%) were elderly, and 48.57% of TB patients were asymptomatic. Nearly 50% patients did not present cough of any duration, and less than half present cough more than 2 weeks, a defined suspected symptom in China NTP. Cough of any duration was reported more in patients aged under 65 than those in elderly, especially for the acute cough (9.78% vs 6.87%). Those symptoms defined by China NTP were reported by less than half participants in two subpopulations. Acute cough (<2 weeks) was an independent predictor of TB in people aged under 65 (adjusted OR: 3.3, 95% CI: 2.0-5.5), but not in those aged 65 and above (adjusted OR: 1.4, 95% CI: 0.7-2.9). The specificity for each symptom was significantly higher in participants aged under 65 (P<0.01), and sensitivities of most symptoms were significantly higher among elderly (P<0.05 or P<0.01). When compared with cough for 2 weeks and more, using cough of any duration for symptom screening increased the sensitivity from 42.9% to 51. % for all participants, and the AUC increased from 0.70 to 0.74 for participants aged under 65 without significant difference.

**Conclusions:**

There is a high percent of asymptomatic TB patients, and those symptoms adopted in China NTP for screening is poorly predictive for TB. The presence of TB symptoms, the sensitivities and specificities of symptoms for TB were distinct between two subpopulations cut by age 65, implying different case finding strategies should be established for them. The current case finding strategy should be improved, and further studies should be done to evaluate the performance and cost-effectiveness of different symptom screening strategy.

## Introduction

In 2013, an estimated totally 9 million people developed TB, within which about 3 million cases were missed by national surveillance system [1]. The routine TB surveillance system in China covered all TB dispensaries and general hospitals nationwide, and the notification rate was very close to TB incidence rate estimated by World Health Organization (WHO) [2]. However, there was a big gap between the number of TB cases notified in surveillance system and that estimated by WHO because of our large population. In 2013, China had an estimated 0.98 million incident TB cases, and only 0.86 million cases notified in surveillance system [1].

Tuberculosis Management Information System is relatively perfect in China, and the reason why there were still so many missed cases in China is closely related to the current passive-case finding strategy [3]. Almost all TB patients notified by the routine surveillance system were those who voluntarily present themselves to health-care facilities because of suspected TB symptoms. TB patients without suspected symptoms and those who present symptoms but don’t visit health-care facilities could not get timely diagnosis and thus be missed by surveillance system. Symptom screening for TB(mostly passive) was a key component of DOTS strategy for combating TB, and the health care provider-initiated active TB screening in high-risk population had become an important strategy recommended by WHO[4]. The active case-finding in China had been implemented in close contacts of sputum smear positive TB patients and the main measurement was based on suspected symptom screening. Although there were several symptoms for TB (such as cough longer than 2 weeks, haemoptysis, low grade fever, weight loss, night sweat and others), the definition of suspected symptom in China NTP was cough, expectoration last for longer than 2 weeks or haemoptysis[3]. That means the suspected TB symptom screening was the first step for TB case finding, and those with TB symptom defined by China NTP would accept the followed X-ray and sputum smear examination in routine practice in China. However, symptom screening alone had a low sensitivity and specificity, and even smear-positive TB cases might be missed by provider-initiated symptom screening [5–7].

A national TB prevalence survey had been conducted in China in 2010. All eligible people aged 15 years or older living in the selected sites had received symptom screening and chest radiograph. For those with suspected symptoms or abnormal chest radiograph, sputum smear and culture were followed. In this study, we evaluated the diagnostic value of symptom screening implemented in China NTP for bacteriologically positive TB patients detected in the prevalence survey and whether symptom screening conducted will affect the diagnostic values for elderly and younger people, to provide evidence for further improving TB case finding strategy in China NTP

## Methods

### Ethical Approval

The prevalence survey protocol was reviewed and approved by the Tuberculosis Operational Research Ethics Review Committee of Chinese Ministry of Health, and the need for individual informed consent was waived by the committee.

### Participants and Diagnostic Algorithm

Details of survey procedure and diagnostic algorithm have been described fully elsewhere [8]. Briefly, a population-based survey was conducted in 176 investigation sites nationwide from April to July in 2010, and 252 940 participants were asked to answer a questionnaire and received chest radiograph examination. Three sputum specimens (one “spot” specimen, one early morning specimen, and one night specimen) would be required for those with suspected symptoms or abnormal chest radiograph, and sputum smear, culture and strain identification would be carried out for diagnosing TB.

### Screened TB symptoms and questionnaire survey

In our questionnaire, the presence and duration of cough and haemoptysis that are possibly suggestive of TB included in Chinese NTP were collected. Duration of cough and haemoptysis were recorded in weeks, including shorter than 2 weeks (acute), longer than 2 weeks but shorter than 3 weeks, longer than 3 weeks. Those with cough for longer than 2 weeks (chronic) or hemoptysis were classified as having symptoms of TB.

The questionnaire survey was carried out in eligible participants face to face by trained staff in county level, and data management specialist checked every questionnaire for its completeness and logicality, and revised information on second day. After completing data input, 5% of questionnaires were selected randomly to be checked by national expert group.

### Definition for TB suspects and bacteriologically positive TB

A TB suspect was defined as an individual with TB symptom or abnormal chest radiograph. The abnormality of chest radiograph was identified during field reading by trained clinic doctor and radiological doctor, and a national expert group reviewed all chest radiograph for each TB case.

A bacteriologically positive TB patient was defined as an individual with at least one positive smear or culture. Positive smear had at least one acid-fast bacillus identified within 100 fields under microscopy, and positive culture had at least one colony of *Mycobacterium tuberculosis* complex isolated by using Löwenstein-Jensen medium.

The positive smear TB was assessed and classified strictly by trained laboratory technician in county level according to standardized methods and procedure, and the quality of culture conducted in county or city laboratory was monitored by provincial tuberculosis reference laboratory or the National Tuberculosis Reference Laboratory.

### Statistical analysis

Data was analyzed by using SPSS Statistics 17.0. Only newly diagnosed bacteriologically positive TB were included in our analysis, that is, the prevalent TB cases diagnosed before survey were excluded from analysis for TB patients and for all eligible participants.

We used data from our survey to compare the performance of the suspected symptom of any duration with that of the current algorithm implemented in China NTP for two subpopulations divided by age 65. The OR and 95% confidence intervals (CIs) for various symptoms were examined in single variable analysis and in a multiple logistic regression model adjusted for gender, age, TB history and other screened symptoms. The sensitivity, specificity, PPV and NPV of screened symptoms for TB were calculated. AUC and 95% CIs were also estimated by using ROC curve analysis to provide a summary measure of diagnostic accuracy, and a non-parametric test was used to compare different sensitivities, specificities and AUCs.

## Results

### Participants and patients

Of the totally 252 940 participants who consent to TB screening in our survey, 146 were already diagnosed as active TB before survey, leaving 252 794 participants for this analysis. Following the diagnostic protocol, 315 individuals were diagnosed as bacteriologically positive TB.

The median age of the newly diagnosed bacteriologically positive TB patients was 61 years (range, 15–88), and 131 patients (42%) were elderly. Of these patients, 227 cases (72%) were male. The gender and age specific distribution was presented in Table 1.

**Table 1:** The Percentages of Bacteriologically Positive TB Patients, by Gender and Age Status, China, 2010.

Age groups	Male		Female		All patients	
	No.	%	No.	%	No.	%
15-	2	0.88	2	2.27	4	1.27
20-	7	3.08	2	2.27	9	2.86
25-	7	3.08	6	6.82	13	4.13
30-	6	2.64	3	3.41	9	2.86
35-	17	7.49	4	4.55	21	6.67
40-	8	3.52	9	10.23	17	5.40
45-	17	7.49	6	6.82	23	7.30
50-	13	5.73	6	6.82	19	6.03
55-	31	13.66	4	4.55	35	11.11
60-	26	11.45	8	9.09	34	10.79
65-	18	7.93	7	7.95	25	7.94
70-	35	15.42	16	18.18	51	16.19
75-	28	12.33	11	12.50	39	12.38
80-	10	4.41	1	1.14	11	3.49
85-	2	0.88	3	3.41	5	1.59
Total	227	100.00	88	100.00	315	100.00

### The distribution of TB symptoms

The distribution of different TB symptoms among survey participants and bacteriologically confirmed cases are shown in Table 2. Most of the screened TB symptoms were significantly more common in elderly than those in aged under 65. Overall, 7.17% of participants aged under 65 and 13.88% of elderly had any duration of cough or haemoptysis (P<0.01). According to those symptoms defined by China NTP, the suspected symptoms were reported in 1.64% of participants aged under 65 and 4.94% of elderly, respectively (P<0.01).

**Table 2:** Prevalence Rates of TB Symptoms in Survey Participants and Bacteriologically Positive TB Patients, by Age Status, China, 2010.

Presence of	All survey participants				All bacteriologically TB patients			
	Total (n = 252794) (%)	No. participants aged under 65 (n = 214807) (%)	No. participants aged 65 and above (n = 37987) (%)	P value	Total (n = 315)(%)	No. patients aged under 65 (n = 184) (%)	No. patients aged 65 and above (n = 131) (%)	P value
Cough (with or without haemoptysis)								
any duration	20643 (8.17)	15380 (7.16)	5263 (13.85)	<0.01	162 (51.43)	95 (51.63)	67 (51.15)	0.09
<2 weeks	15351 (6.07)	11941 (5.56)	3410 (8.98)	<0.01	27 (8.57)	18 (9.78)	9 (6.87)	0.11
2–3 weeks	1901 (0.75)	1289 (0.60)	612 (1.61)	<0.01	29 (9.21)	18 (9.78)	11 (8.40)	0.15
≥3 weeks	3391 (1.34)	2150 (1.00)	1241 (3.27)	<0.01	106 (33.65)	59 (32.07)	47 (35.88)	0.07
Haemoptysis (with or without cough)								
any duration	288 (0.11)	206 (0.10)	82 (0.22)	<0.01	14 (4.44)	8 (4.35)	6 (4.58)	0.22
<2 weeks	63 (0.02)	49 (0.02)	14 (0.04)	>0.05	1 (0.32)	1 (0.54)	0 (0.00)	0.58
2–3 weeks	54 (0.02)	39 (0.02)	15 (0.04)	<0.01	3 (0.95)	2 (1.09)	1 (0.76)	0.43
≥3 weeks	171 (0.07)	118 (0.05)	53 (0.14)	<0.01	10 (3.17)	5 (2.72)	5 (3.82)	0.22
Cough and haemoptysis								
any duration	251 (0.10)	178 (0.08)	73 (0.19)	<0.01	14 (4.44)	8 (4.35)	6 (4.58)	0.22
<2 weeks	61 (0.02)	47 (0.02)	14 (0.04)	>0.05	1 (0.32)	1 (0.54)	0 (0.00)	0.58
2–3 weeks	38 (0.02)	29 (0.01)	9 (0.02)	>0.05	3 (0.95)	2 (1.09)	1 (0.76)	0.43
≥3 weeks	152 (0.06)	102 (0.05)	50 (0.13)	<0.01	10 (3.17)	5 (2.72)	5 (3.82)	0.22
Cough or haemoptysis of any duration	20681 (8.18)	15409 (7.17)	5272 (13.88)	<0.01	162 (51.43)	95 (51.63)	67 (51.14)	0.09
Suspected symptoms defined by China NTP	5390 (2.13)	3514 (1.64)	1876 (4.94)	<0.01	136 (43.17)	78 (42.39)	58 (44.27)	0.09

For newly diagnosed bacteriologically positive TB patients, although presence of symptoms differed a little between two subpopulations, the difference was not statistically significant. Cough of any duration was reported more in patients aged under 65 than elderly, especially for the acute cough (9.78% vs 6.87%). Overall, the percentage of asymptomatic TB patients hit 48.57%, and the prevalence rates for any duration of cough or haemoptysis were 51.63% for patients aged under 65 and 51.14% for elderly, however, the symptoms defined by China NTP were reported by 42.39% and 44.27% of these two subpopulations respectively.

Of the 315 newly diagnosed bacteriologically positive TB cases, 136 patients (43.17%) reported that they had symptoms defined by China NTP, leaving 56.83% of bacteriologically positive TB cases missed by NTP hypothetically, with percentages of missed patients being 57.61% in participants aged under 65 yrs and 55.72% in elderly.

### The association of symptom with bacteriologically positive TB

In general, TB patients tended to be polysymptomatic and the presence of symptoms was affected by other factors. A multiple logistic regression model was used to identify the specific screened symptom independently associated with bacteriologically positive TB diagnosis. As shown in Table 3, for each symptom, ORs adjusted for gender, age, TB history and other suspected symptoms tended to be much lower than unadjusted ORs. For participants aged under 65, acute cough (adjusted OR: 3.3), chronic cough (adjusted OR: 29.3 for duration of 2–3 weeks and 53.2 for duration of more than 3 weeks), haemoptysis (adjusted OR: 3.2) remained independent predictors of bacteriologically positive TB diagnosis. However, the presence of acute cough was not associated with bacteriologically positive TB diagnosis among elderly (adjusted OR: 1.4, 95% CI: 0.7–2.9).

In addition, adjusted ORs for the same symptom were much higher in participants aged under 65 than those in elderly.

**Table 3:** Association of Screened Symptoms and Bacteriologically Positive TB, by Age Status, China, 2010 (n = 315).

Screened symptoms	Participants aged under 65		Participants aged 65 and above	
	unadjusted ORs (95%CI)	Adjusted ORs (95%CI)	unadjusted ORs (95%CI)	Adjusted ORs (95%CI)
Cough or haemoptysis of any duration				
No	1	1	1	1
Yes	22.6(16.9–30.2)	13.3(9.8–18.1)	8.3(5.9–11.8)	6.9(4.8–9.8)
Cough (with or without haemoptysis)				
No	1	1	1	1
<2 weeks	5.5(3.3–9.2)	3.3(2.0–5.5)	1.7(0.8–3.5)*	1.4(0.7–2.9) *
2–3 weeks	51.6(31.0–85.9)	29.3(17.4–49.6)	11.8(6.2–22.5)	9.3(4.8–17.8)
≥3 weeks	101.5(72.8–141.5)	53.2(37.1–76.3)	25.2(17.2–36.9)	18.4(12.3–27.6)
Haemoptysis (with or without cough)				
No	1	1	1	1
Yes	77.5(37.7–159.6)	3.2(1.5–6.7)	28.5(12.2–66.5)	3.1(1.3–7.6)
Suspected symptom defined by China NTP				
No	1	1	1	1
Yes	71.4(53.2–95.9)	41.3(30.2–56.4)	19.4(13.7–27.5)	15.6(11.0–22.3)

CI = Confidence interval

* P value > 0.05

### The performance characteristics of TB symptoms for bacteriologically positive TB diagnosis

Measure of the performance of different screened symptoms for bacteriologically positive TB diagnosis in subpopulations were presented in Table 4. Although age status did not have a significant influence on AUC (P>0.05), the specificities of all screened symptoms were higher in participants aged under 65 than those in elderly (P<0.01), and sensitivities for most symptoms were lower among them (P<0.05 or P<0.01), except for any duration of cough/cough or haemoptysis. In addition, PPVs were considerably higher in elderly than those in aged under 65.

**Table 4:** Performance Characteristics of Screened Symptom for Bacteriologically Positive TB, by Age Status, China, 2010.

Screened symptoms	No. of participants with defined symptoms	No. of TB cases with defined symptoms	Sensitivity	Specificity	PPV	NPV	AUC
Cough or haemoptysis of any duration							
Participants aged under 65	15409	95	51.6	95.5	0.6	99.9	0.74
Participants aged 65 and above	5272	67	51.1	88.8**	1.3	99.8	0.70
All participants	20681	162	51.4	94.7	0.8	99.9	0.73
Cough of any duration							
Participants aged under 65	15380	95	51.6	95.5	0.6	99.9	0.74
Participants aged 65 and above	5263	67	51.1	88.9**	1.3	99.8	0.70
All participants	20643	162	51.4	94.7	0.8	99.9	0.73
Cough ≥2 weeks							
Participants aged under 65	3439	77	41.8	99.0	2.2	99.9	0.70
Participants aged 65 and above	1853	58	44.3**	96.1**	3.1	99.8	0.70
All participants	5292	135	42.9	98.7	2.5	99.9	0.71
Cough ≥3 weeks							
Participants aged under 65	2150	59	32.1	99.4	2.7	99.9	0.66
Participants aged 65 and above	1241	47	35.9**	97.4**	3.7	99.8	0.67
All participants	3391	106	33.7	99.1	3.1	99.9	0.66
Haemoptysis							
Participants aged under 65	206	8	4.3	99.9	3.8	99.9	0.52
Participants aged 65 and above	82	6	4.6*	99.8**	7.1	99.7	0.52
All participants	288	14	4.4	99.9	4.8	99.9	0.52
Suspected symptom defined by China NTP							
Participants aged under 65	3514	78	42.4	99.0	2.2	99.9	0.71
Participants aged 65 and above	1876	58	44.3**	96.1**	3.0	99.8	0.70
All participants	5390	136	43.2	98.6	2.5	99.9	0.71

PPV, positive predictive value; NPV, negative predictive value; AUC, area under the receiver operating characteristic curve

In analysis of cough and haemoptysis, the AUC for any duration of cough were significantly larger than for haemoptysis (P<0.01 for both subpopulations)

When analysis was conducted in participants under aged 65, the sensitivities of cough of any duration/ Cough or haemoptysis of any duration were significantly higher than that of suspected symptom defined by China NTP (P<0.01).

* Compared with participants aged under 65, P<0.05

** Compared with participants aged under 65, P<0.01

When compared cough with haemoptysis, the sensitivity of cough was much higher than that of haemoptysis in both subpopulations, and the difference of AUC was statistically significant (P<0.01 in both groups). When compared with cough for 2 weeks and more, cough of any duration increased the sensitivity from 42.9% to 51.4% for all participants. And, although there was no significant difference, the AUC for cough of any duration increased from 0.70 to 0.74 for participants aged under 65.

## Discussion

Our study assessed the diagnostic accuracy of symptom screening for TB implemented in China NTP in a nation-representative population survey, and provided evidence for improving the future TB case finding strategy.

Passive case finding was the routine practice in China for finding TB patients, and persistent cough was the main symptom for initiating TB examination. The most striking finding in our study was nearly 50% bacteriologically confirmed TB patients did not present cough of any duration, and less than half of those patients present cough more than 2 weeks. Those two percentages are consistent with those reported in Vietnam and those for people with HIV[5,9,10]. In our study, only about 43% of patients met the criteria of suspected symptoms defined by China NTP, and the sensitivity was about 43%, implying many patients missed by routine practice. Several investigations carried out in people living with HIV found that asking them only about chronic cough was an insensitive approach for screening TB, and a combination of symptoms could increase the sensitivity largely to 79%-93% among people living with HIV[10–13]. The result from our study suggested the definition of TB suspected symptom currently used in China NTP should be improved, and more symptoms should be included.

Although being elderly or not did not present significant influence on the performance characteristics of symptom screening for TB diagnosis in our study, it was notable that there were differences in ORs, sensitivities and specificities of symptoms for detecting TB between participants aged under 65 and elderly. For cough, the most common symptom for TB, those adjusted ORs for different durations are more than two times higher in those aged under 65. Acute cough is still an independent predictor of TB adjusted for haemoptysis and other factors among participants aged under 65. Like other studies[5,10], the sensitivity of cough increased from 41.8% when chronic cough was used to define TB suspect to 51.6% when cough of any duration was used among elderly, the AUC accordingly increased slightly from 0.70 to 0.74. Meanwhile, a high prevalence rate of cough of any duration (7.16%) was observed in our survey, and a vast majority of them were acute cough. The percentage of patients with acute cough was higher in TB patients aged under 65 (9.78%) than that in elderly (6.87%). These findings from our study suggested expanding the current symptom for population aged under 65 would increase the yield of case finding in routine practice.

Poor performance of symptom screening alone for detecting TB had been found in several studies, even the higher sensitivity of four-part symptom screen, including any of cough, fever, night sweats, and weight loss, was still unsatisfying[5,10,13–15]. Symptom screening was attractive because it was simple, does not need expensive equipment, and has been applied in several surveys[16–18]. However, compared with symptom screening alone, chest X-ray (CXR) examination alone had higher accuracy, and combined CXR and symptom screening had the highest sensitivity[7,12,14]. Active TB case finding was a very costly endeavor, and was cost-effective only if the TB prevalence among the target population was high[19]. WHO strongly recommended systematic active case finding should be considered among people who belong to selected high risk groups[20]. Elderly people was a confirmed high risk group for TB, and TB prevalence among them in China hit about 1 000/100 000[21], making active case finding in elderly would enhance the yield of TB identification. The standard for National Project of Basic Public Health Service launched by Ministry of Health in 2011 provided a good chance for making active case finding in elderly. In this project, a regular physical examination was provided for elderly yearly, and in some developed regions, CXR examination had been added into this service package, by which active case finding in this high risk group is feasible. Based on the 55.72% of patients missed in elderly resulted from our survey, there would be an estimated 1.2 times increases for number of TB patients identified by active case finding strategy compared with that found by current NTP in elderly group.

Our study has several strengthens. Firstly, we used a population based sample randomly selected nationwide by using multistage, stratified sampling. Secondly, we used bacteriologically confirmed TB rather than active pulmonary TB, by which avoiding the bias resulted from uncertainty of clinical diagnosis for TB. Finally, we conducted strict quality control for data collection, chest radiograph and lab test, by which the survey quality could be guaranteed and bias could be declined.

Our study did have some limitations. Firstly, individuals younger than 15 years old had been excluded from our survey. The prevalence rate of symptom might be different among those children and likely to be more TB-specific, thus, how to change case finding strategy among this group would lack evidence due to absence of performance evaluation of symptom screening for TB. Secondly, the questionnaire we used included a limited range of questions. Only information of cough and haemoptysis, the defined suspected TB symptoms in China NTP, was collected. Other symptoms that were possibly suggestive of TB, such as fever, night sweat and weight loss, were excluded from our survey, with a reduction of diagnostic sensitivity. Meanwhile, incomplete or inaccurate symptom reporting by participants potentially existed in population based survey, although we carried out strict quality control measurement. Thirdly, considering symptom screening was the first step for determining whether CXR and sputum smear should be done in current China NTP, and CXR are more costly and not as easy to conduct as symptom interview for screening, although CXR could increase the sensitivity of diagnosis, its value was excluded in our current analysis. Finally, information of other factors (including history of diabetes and other diseases, smoking status, body mass index, and others) were absent from our questionnaire, resulting in failure to analyze the performance of TB symptom in population with multi risk factors and to provide evidence for ascertaining the target population for making active case finding in limited-resource areas.

## Conclusions

This study has shown a high percent of asymptomatic TB patients and a poor performance of symptom screening implemented in China NTP for TB case finding. Increases in sensitivity and AUC of using duration expanded cough as screening symptom in participants aged under 65 years old points to the need to dramatically lower the threshold for TB symptom screening. And, a large increase in number of patients identified in elderly could be predicted by integrating active case finding practice into the national project.

Further studies are needed. For the current case finding strategy in China NTP, evaluation of the performance of combined CXR and symptom screening should be done, to identify the change of screening yield in different procedures, helping to develop an algorithm with higher sensitivity and specificity. Cost-effective analysis for current and other improved screening approaches should be conducted too to provide reference for policy making. Futhermore, new diagnostic tools, such as LED, GeneXpert and immunological test etc., should be evaluated for their screening value, to enlarge the case finding yield.
